# Ionic Origin of Electro-osmotic Flow Hysteresis

**DOI:** 10.1038/srep22329

**Published:** 2016-02-29

**Authors:** Chun Yee Lim, An Eng Lim, Yee Cheong Lam

**Affiliations:** 1 School of Mechanical and Aerospace Engineering, Nanyang Technological University, Nanyang Avenue 50, 639798, Singapore

## Abstract

Electro-osmotic flow, the driving of fluid at nano- or micro- scales with electric field, has found numerous applications, ranging from pumping to chemical and biomedical analyses in micro-devices. Electro-osmotic flow exhibits a puzzling hysteretic behavior when two fluids with different concentrations displace one another. The flow rate is faster when a higher concentration solution displaces a lower concentration one as compared to the flow in the reverse direction. Although electro-osmotic flow is a surface phenomenon, rather counter intuitively we demonstrate that electro-osmotic flow hysteresis originates from the accumulation or depletion of pH-governing minority ions in the bulk of the fluid, due to the imbalance of electric-field-induced ion flux. The pH and flow velocity are changed, depending on the flow direction. The understanding of electro-osmotic flow hysteresis is critical for accurate fluid flow control in microfluidic devices, and maintaining of constant pH in chemical and biological systems under an electric field.

Electro-osmotic flow (EOF) or electro-osmosis is the flow of fluid in a micro-/nano-sized channel or a porous material under an externally applied electric field. EOF arises due to the spontaneous charge formation at the liquid-solid interface. Typically, negative charges are formed naturally on a solid surface when it is placed in contact with water or aqueous solutions. The positive ions in the liquid are then attracted to the negatively-charged surface while the negative ions are repelled from it, forming a thin layer of net charges called electrical double layer (EDL). When an electric field is applied parallel to the solid surface, the positively-charged EDL experiences a body force and moves in the direction of the electric field. The movement of EDL is transferred to the bulk of the liquid through viscous effect to generate EOF. If the EDL is thin as compared to the size of the channel, the fluid flow velocity *U* is given by the Helmholtz-Smoluchowski slip velocity equation: *U *= *−ε*_*o*_*ε*_*r*_*Eζ* /*μ*, where *E* is the electric field, *ε*_*o*_ is the permittivity of free space, *ε*_*r*_ is the relative permittivity of the liquid, *ζ* is the zeta potential (electric potential developed at the solid surface) and *μ* is the viscosity of the liquid.

EOF has been exploited in various applications including drug delivery^1^,^2^, fuel cell^3^,^4^, sludge treatment^5^,^6^, deoxyribonucleic acid (DNA) focusing and manipulation^7^,^8^, disease diagnosis from blood^9^, chemical species separation^10^, pumping^11^ and mixing of fluids^12^,^13^ in various microfluidic devices. In many of these applications, the fluid driven by EOF is inhomogeneous, differing in conductivity and concentration. It has been experimentally observed that EOF exhibits hysteretic behavior when the flow involves the displacement of two fluids with different concentrations^14^ or ionic species^15^ in a microchannel. Contrary to the prediction of prevailing EOF theory, the flow behavior is found to be different, and dependent on the flow direction. For example, the flow of a lower concentration displacing higher concentration potassium chloride (KCl) solutions is slower than the flow in the reverse direction^14^. Hitherto, the origin for such hysteretic effect is not well understood.

Ionic distributions during EOF in nanochannels have been examined with molecular dynamics simulations^16^,^17^,^18^. However, the effect of conductivity or ion concentration gradient on EOF has not been considered in these investigations. Previous investigations on EOF involving solutions with different concentrations^19^,^20^,^21^,^22^ only focus on the behavior of the main constituent ionic species (such as K^+^ and Cl^−^ ions in KCl solutions) and fail to capture the main effect of EOF hysteresis. Similarly, the conventional analyses of EOF in microchannel with heterogeneous zeta potential^23^,^24^ only consider the distribution of main constituent ionic species.

As EOF is a surface-driven phenomenon, it is only intuitive to examine surface-related effects for the cause of EOF hysteresis. Contrary to this obvious notion, we demonstrate in this investigation that EOF hysteresis originates from the ionic behavior in the bulk of the fluid. Even more surprising, it is the minority ions, such as hydronium ions (H_3_O^+^) in aqueous solutions that induce EOF hysteresis. The understanding of EOF hysteresis is critical to the accurate manipulation of fluids and solutes in real world applications, where the fluids involved are typically inhomogeneous.

## Results

### Experimental results

EOF hysteresis was demonstrated and quantified by monitoring the current change during the displacement flow of two aqueous solutions with different ion concentrations in a glass microcapillary (see Methods section). As the solution with a different conductivity (due to the difference in ion concentration) flows into the capillary, the solution which initially resides in it is displaced and the total resistance of the capillary is changed. The change of resistance is captured as the current change, from which the flow behavior can be examined. When the displacement process is completed, the current becomes constant and the time to reach a steady current is termed the displacement time. The details for the determination of displacement time from the experimental current-time curve can be found in Supplementary Note 1.

We hypothesized that EOF hysteresis is caused by the presence of pH-governing minority ions which are typically neglected in conventional EOF studies. The mechanics of EOF hysteresis was examined through the displacement flow of three dilute ionic solutions, namely KCl, sodium bicarbonate (NaHCO_3_) and potassium dihydrogen phosphate (KH_2_PO_4_), with concentration of 0.2 mM and 1 mM. KCl is selected as a model for a simple salt solution. The concentration is set to 0.2–1 mM range to minimize Joule heating effect which might change the temperature and other properties of the solution during the experiment (see Methods section for more details). NaHCO_3_ and KH_2_PO_4_ are selected because the anions (HCO_3_^−^ and H_2_PO_4_^−^) are pH buffering ion species. These buffer solutions have been employed to verify that pH change is responsible for causing EOF hysteresis.

**Figure 1:**
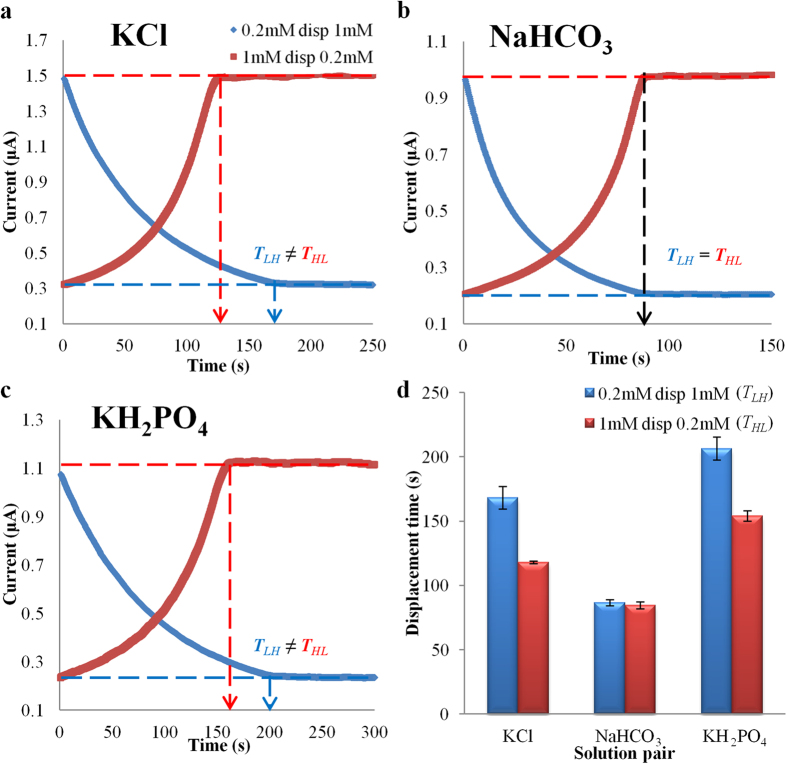
Experimental results demonstrating EOF hysteresis. Current-time curves obtained from monitoring current changes during the displacement flow of 0.2 mM and 1 mM for (**a**) KCl (**b**) NaHCO_3_ (**c**) KH_2_PO_4_ solutions. (**d**) Displacement time difference in two directions for KCl, NaHCO3 and KH_2_PO_4_ solution pairs. *T*_*LH *_= time for low concentration solution to displace high concentration solution. *T*_*HL *_= time for high concentration solution to displace low concentration solution.

Figure 1a shows that for 1 mM KCl to displace 0.2 mM KCl, the displacement time (*T*_*HL*_) was approximately 30% less than the time (*T*_*LH*_) for the displacement flow in the reverse direction. However, the cause of this hysteretic behavior is not well understood as it defies conventional understanding of EOF. Based on the Helmholtz-Smoluchowski equation, the EOF flow velocity is proportional to electric field. The applied electric field distribution across the microchannel is dependent on ion concentration distribution, which dictates the electrical resistance (see Equation S8 in Supplementary Note 2). When the applied electric field and EOF are reversed, one would expect that the ions will retrace the same trajectories as the forward flow, with the same flow time. However, the experimental results have shown that the flow rates for the two directions are indeed different.

The same experiment was repeated with 1 mM and 0.2 mM NaHCO_3_ solutions. The hysteretic behavior vanished: the displacement times for both directions are identical (see Fig. 1b). Another set of experiment was conducted with KH_2_PO_4_ solution pair. The displacement time difference for the two flow directions are approximately 25% (see Fig. 1c), slightly lower than that of KCl solution pair. Both NaHCO_3_ and KH_2_PO_4_ consist of ion species which can act as pH buffers, namely bicarbonate ions HCO_3_^−^ and dihydrogen phosphate ions H_2_PO_4_^−^. It is hypothesized that the pH-governing ions such as hydronium ions (H_3_O^+^) and hydroxide ions (OH^−^) are the culprits for EOF hysteresis. In a typical ionic solution, these ions are minority species, which are a few orders of magnitude smaller in concentration as compared to the main constituent ions of the solution. For example, in 1 mM KCl solution, the concentrations of H_3_O^+^ and OH^−^ are 2.07 μM and 4.83 nM respectively. Although this investigation indicates that they should not be, but they are typically neglected in conventional theoretical formulation of EOF.

### Origin of electro-osmotic flow hysteresis

**Figure 2:**
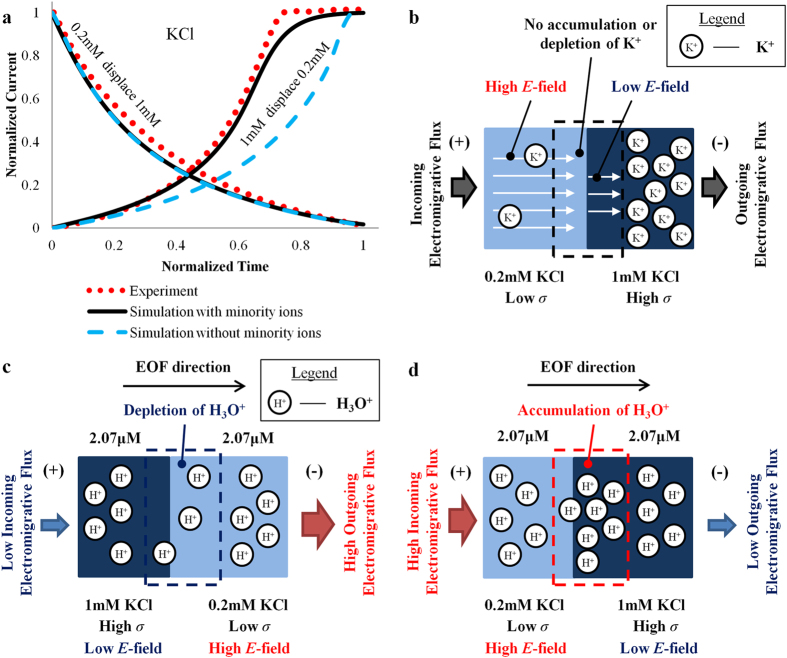
Depletion and accumulation of H_3_O^+^ ions. (**a**) Current-time plot for displacement flow of 0.2 mM and 1 mM KCl. (**b**) Flux balance of K^+^ ions (main constituent ions) in displacement flow of 0.2 mM and 1 mM KCl (**c**) Depletion and (**d**) accumulation of hydronium (H_3_O^+^) ions (minority ions) due to imbalance of electromigrative flux across the interface of 0.2 mM and 1 mM KCl solution. *E*-field = electric field; *σ *= conductivity of solution.

Numerical simulations were performed to elucidate the mechanics of EOF hysteresis. A new EOF model which includes the pH-governing minority ions, including H_3_O^+^, OH^−^ and HCO_3_^−^ along with their acid-base equilibria has been developed. HCO_3_^−^ ions exist in KCl solution as carbon dioxide (CO_2_) in air dissolves in water (refer to carbonate acid-base equilibria in Supplementary Note 2). To verify that these minority pH-governing ions are indeed responsible for EOF hysteresis, another set of simulations based on the conventional EOF theory without minority ions have also been conducted as a comparison to our numerical model. The conventional simulation without minority ions predicts similar displacement time in both flow directions (see Fig. 2a), i.e. hysteretic effect is absent. The simulation results based on conventional EOF model deviates significantly from the experimental results. In contrast, our new model which includes minority ions shows good agreement with experimental results (see Fig. 2a), demonstrating the existence of EOF hysteresis.

To understand the mechanics of EOF hysteresis, comprehension on the distinction between the behaviors of majority and minority ions is critical. The migration flux of ionic species induced by electric field (which is termed as electromigrative flux) is proportional to its concentration *c*_*i*_ and the electric field strength *E*. The electromigrative flux is given by *c*_*i*_*u*_*m(i)*_*E*, where *u*_*m(i)*_ is the ionic mobility of each species *i*. The solution with a lower conductivity experiences a higher electric field, and vice versa. This is given by the expression *E *= *J*/*σ*, where *J* is the current density and *σ* is the conductivity of solution. However, the conductivity of a solution is proportional to its ion concentration and given by *F* ∑*z*_*i*_*u*_*m(i)*_*c*_*i*_, where *F* is the Faraday constant and *z*_*i*_ is the ion charge number of each species *i*. Therefore, the electromigrative flux of main constituent species *i* for a symmetric electrolyte can be written as:


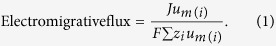


The ion concentration *c*_*i*_ has been cancelled out and the flux is a constant. Therefore, the incoming and outgoing fluxes for the main constituent ions (K^+^ and Cl^−^ in KCl solution for example) will be exactly balanced (see Fig. 2b). Hence, the main constituent ions will never experience accumulation or depletion at the interface between two solutions with different concentration.

However, the same cannot be said for the minority ions (H_3_O^+^, HCO_3_^−^, OH^−^ etc), which have negligible/no influence over the conductivities of the solution. The electric field distribution has been decided by the main constituent ions (which determine the conductivities). These minority ions have been forced to migrate according to the local electric field. Therefore, the incoming and outgoing fluxes of these minority ions at the interface between the two solutions will not be balanced, i.e. there is a spatial gradient of ion flux across the interface. This leads to and causes either the depletion and accumulation of ions.

The pH for 0.2 mM and 1 mM KCl are identical and thus both solutions have the same concentration of H_3_O^+^ ions. When 1 mM KCl displaces 0.2 mM KCl, the incoming flux of H_3_O^+^ is lower than the outgoing flux. H_3_O^+^ ions are depleted at the boundary (concentration decrease, see Fig. 2c). In contrast, when 0.2 mM displaces 1 mM KCl, the incoming flux H_3_O^+^ is higher than the outgoing flux, causing H_3_O^+^ to be accumulated at the interface (concentration increase, see Fig. 2d).

When the H_3_O^+^ ions are depleted or accumulated at the interface, the pH of the solution at the interface is changed. Zeta potential is the electric potential developed at the solid surface due to the formation of EDL, and governs the direction and velocity of EOF. The value of zeta potential is sensitive to the pH in the solution. For this study, glass micro-capillaries were employed as the microchannel. The zeta potential development at glass/silica surface is caused by the deprononation (proton removal) of silanol (SiOH) groups when it is placed in contact with an aqueous solution: SiOH + H_2_O ⇌ SiO^−^ + H_3_O^+^.

If the H_3_O^+^ concentration is increased, the equilibrium will be shifted to the left, resulting in lesser SiO^−^ groups. This effect lowers the concentration of negatively-charged groups on the glass surface and reduces the magnitude of zeta potential. On the contrary, when the concentration of H_3_O^+^ is reduced, the equilibrium is shifted to the right, increasing the concentration of SiO^−^ groups and the magnitude of zeta potential of the surface. Our numerical model relates the concentration of H_3_O^+^ to the reversible chemical reaction of silanol surface group and zeta potential formulation (see charge-regulated Grahame equation in Supplementary Note 2). This allows the model to capture the changes of zeta potential in response to pH changes accurately.

**Figure 3:**
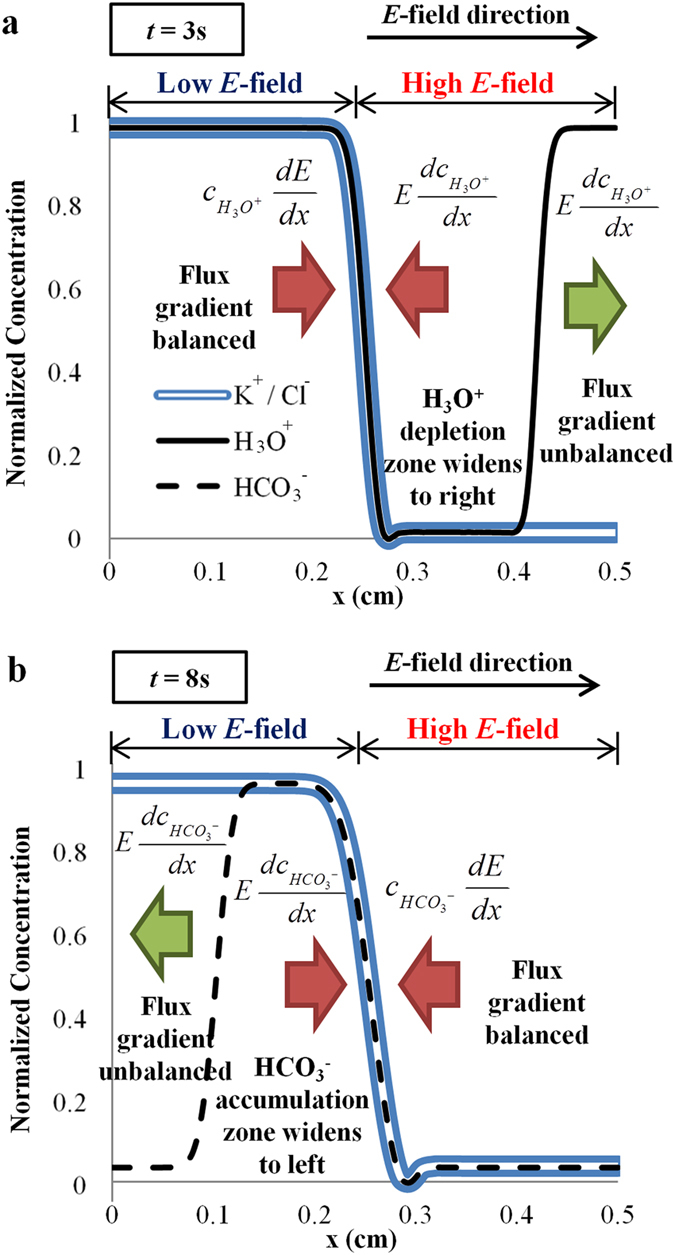
Widening of ion accumulation/depletion zone. Numerical simulations showing imbalance of electromigrative flux gradient widens (**a**) H_3_O^+^ depletion and (**b**) HCO_3_^−^ accumulation zones when 1 mM KCl displaces 0.2 mM KCl. Convective effect and buffering chemical reactions are not included to highlight the role of electromigration. Electromigrative flux gradient is defined as -*u*_*m(i)*_ (*Edc*_*i*_/*dx*^+^ *c*_*i*_*dE*/*dx*) where *u*_*m(i) *_= ionic mobility, *E *= electric field strength, *c*_*i *_= ion concentration, with corresponding subscript of H_3_O^+^ and HCO_3_^−^. The coefficient outside of the bracket is not shown in the figure for conciseness. All ion concentrations are normalized with the maximum and minimum concentrations.

If the accumulation or depletion of H_3_O^+^ ions only occurs at the interface, the change of zeta potential over the entire channel would be negligible when the channel is sufficient long. Interestingly, this accumulation or depletion zone does not stop at the interface, but widens and spread into the bulk of the solution. Figure 3a shows the H_3_O^+^ depletion zone when 1 mM KCl is displacing 0.2 mM KCl. To examine the widening of the depletion zone, the spatial gradient of electromigative flux for ions is separated into two components:


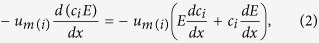


where *x* is the spatial coordinate. The first and second terms on the right of Equation 2 represent the flux gradient due to concentration and electric field gradients respectively.

The initial drop of H_3_O^+^ concentration profile at the interface consists of two edges (see Fig. 3a). The movement of the two edges due to electromigration is examined relative to the interface. Convective effect due to fluid flow and reversible acid-base reactions are neglected. At the left edge, the flux gradient due to concentration and electric field gradients are balanced. Therefore, the left edge is a ‘stationary’ edge which does not move relative to the interface. On the contrary, the right edge has only one component: the electromigrative flux gradient due to concentration gradient. This allows the right edge of H_3_O^+^ depletion zone to move continuously to the right, widening the depletion zone.

Similarly, the negatively charged minority ions experience the widening of accumulation zone in the reverse direction (towards the left, see Fig. 3b). Only HCO_3_^−^ ions (from dissolved CO_2_) are shown here because the concentration of OH^−^ ions is very low for KCl solution with pH 5.68 (see Supplementary Table 1). The concentrations of all the ion species are not independent, but have to obey a set of reversible acid-base equilibria (see Supplementary Note 2). Therefore, the accumulation of HCO_3_^−^ ions also leads to the depletion of H_3_O^+^ ions through the acid-base equilibrium reactions.

**Figure 4:**
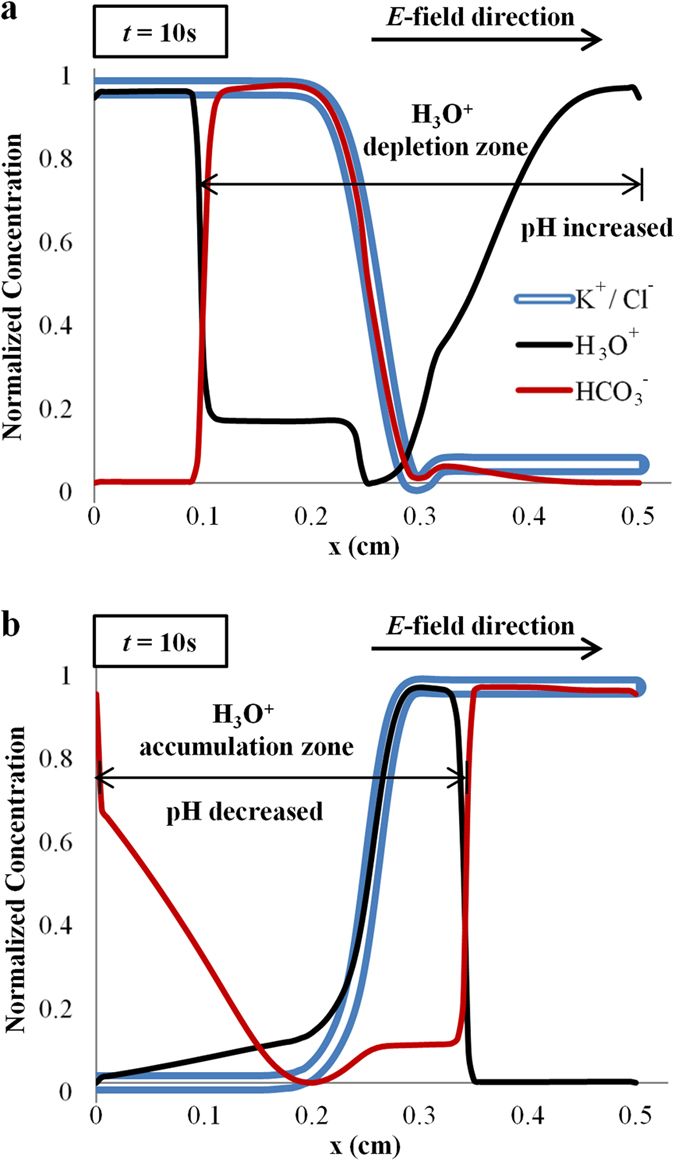
pH changes with widening of ion depletion/accumulation zone. Numerical simulations showing pH changes due to widening of ion accumulation/depletion zone when (**a**) 1 mM displaces 0.2 mM and (**b**) 0.2 mM displaces 1 mM KCl. Concentration of H_3_O^+^ and HCO_3_^−^ ions are related through carbonate acid-base reactions (see Supplementary Note 2). Convective effect is not included. All ion concentrations are normalized with the maximum and minimum concentrations.

For the case of 1 mM KCl displacing 0.2 mM KCl, the pH of 0.2 mM KCl is increased due to the depletion of H_3_O^+^ ions to the right (see Fig. 4a). At the same time, on the left side, the accumulation of HCO_3_^−^ ions (see Fig. 4a) leads to the reduction of H_3_O^+^ as they react to form carbonic acid (H_2_CO_3_) (see Equation S16 in Supplementary Note 2), increasing the pH of 1 mM KCl as well. Consequently, the magnitude of average zeta potential is increased due to an overall higher pH along the microchannel. A larger zeta potential generates a higher flow rate, resulting in a shorter displacement time, as observed experimentally.

When the electric field is reversed (0.2 mM KCl displacing 1 mM KCl), the overall pH is decreased due to the accumulation of H_3_O^+^ ions (see Fig. 4b). The smaller zeta potential is translated to a lower flow velocity and a slower displacement process. The ionic flux imbalance, which leads to pH and zeta potential changes along the microchannel, is the origin of EOF hysteresis.

### Suppression of electro-osmotic flow hysteresis

**Figure 5:**
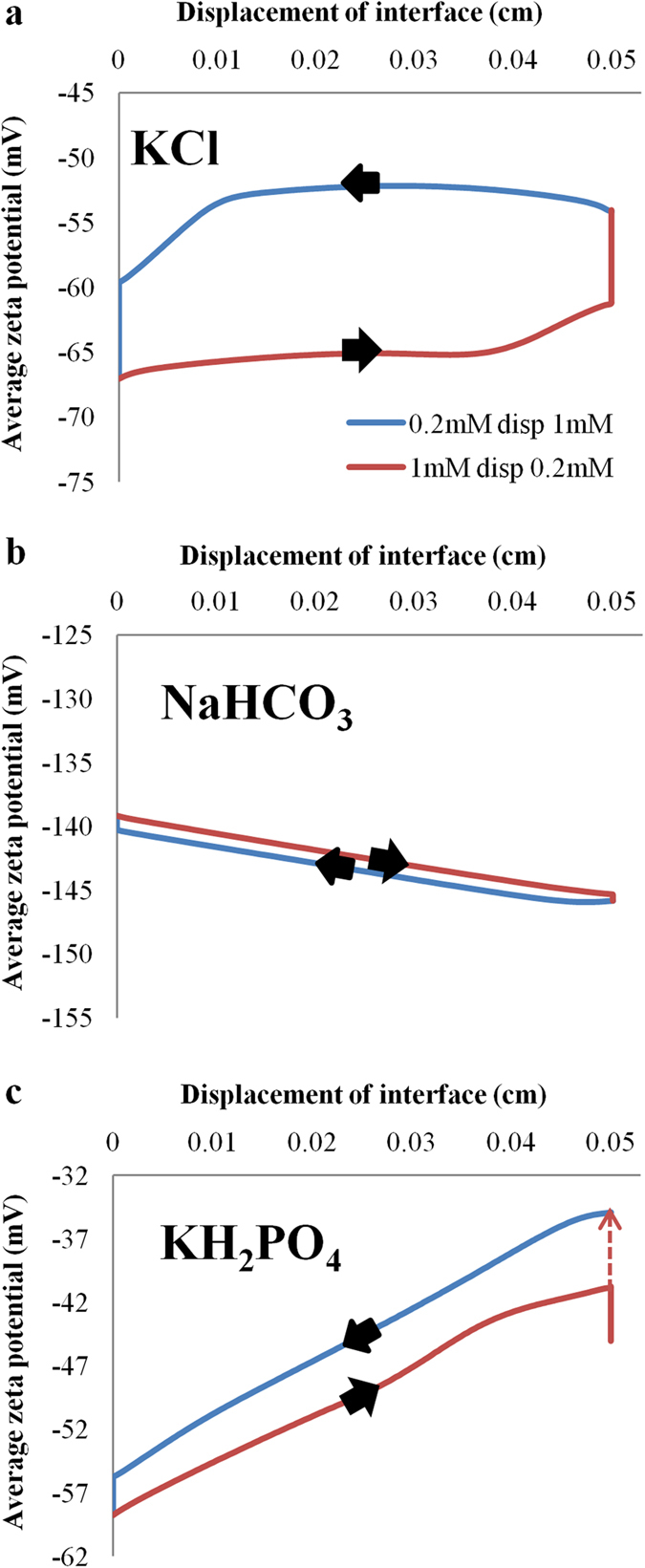
Electro-osmotic flow hysteresis loop. Variations of average zeta potential with interface displacement for (**a**) KCl (**b**) NaHCO_3_ and (**c**) KH_2_PO_4_ solutions in two flow directions. The interface displacement is defined as the position at which the concentration of main constituent ions coincides with the average concentration of the two solutions. The dotted line in (**c**) is drawn to complete the loop because of the excessively long computational time required to complete the loop.

The variations of average zeta potential along the microchannel with interface displacements are shown in Fig. 5. The interface displacement is defined as the position at which the concentration of the main constituent ions coincides with the average concentration of the two solutions. In our case, the interface displacement is the position where the main constituent ion concentration is 0.6 mM which is the average of 0.2 mM and 1 mM. For KCl solution, the zeta potential for the two flow directions follows different paths, forming a hysteresis loop (see Fig. 5a). The hysteresis loop suggests that the zeta potential and EOF flow rate cannot be determined at a given interface position without knowing the flow direction. The two vertical lines at the start and end of the microchannel show that the zeta potential continues to change even when the displacement of the main constituent ions have been completed. This is due to the widen depletion/accumulation zone of pH governing ions.

EOF hysteresis with the resultant pH and zeta potential variations occur as long as there is a conductivity or ion concentration gradient (see Figs 1a, 2a and 5a). It affects the electro-osmotic driven flow rate in microfluidic devices and can even be detrimental to chemical or biological systems which are sensitive to pH variation. To investigate the effect of pH buffering on EOF hysteresis, experiments and numerical simulations with NaHCO_3_ and KH_2_PO_4_ solutions have been performed. The experimental results show that EOF hysteresis vanishes for the displacement flow involving 1 mM and 0.2 mM of NaHCO_3_ (see Fig. 1b). The suppression of EOF hysteresis in NaHCO_3_ solution has been shown in numerical simulations as well (see Supplementary Fig. 1a), with almost identical average zeta potential in both flow directions (see Fig. 5b). However, both experiments and numerical simulations demonstrate that the EOF hysteresis still persists in the displacement flow of KH_2_PO_4_ solutions (see Figs 1c, 5c and Supplementary Fig. 1b). Nonetheless, the displacement time and zeta potential differences have been reduced with the presence of pH buffering ion species.

**Figure 6:**
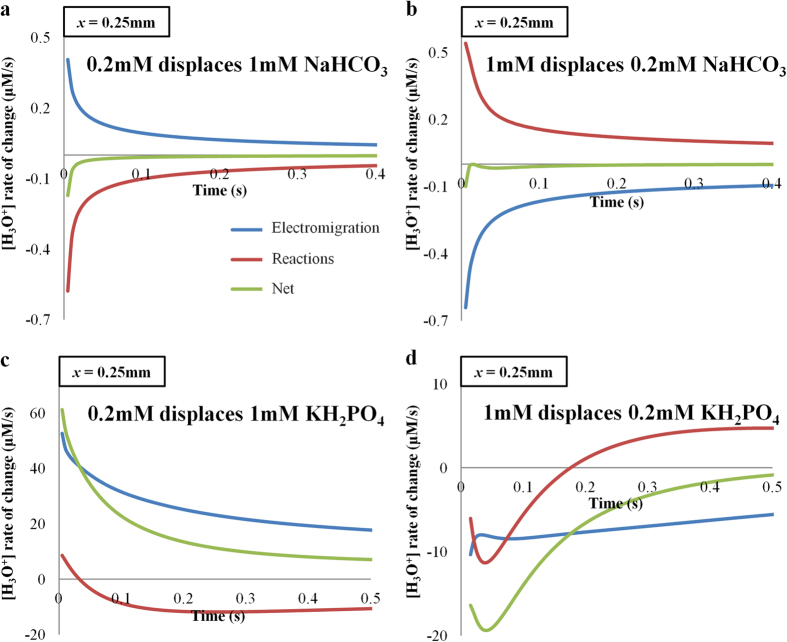
H_3_O^+^ depletion/accumulation rate from electromigration and buffering reactions. Numerical simulations showing rate of change for H_3_O^+^ concentration due to electromigration and acid-base reactions for (**a**) 0.2 mM displacing 1 mM NaHCO_3_, (**b**) 1 mM displacing 0.2 mM NaHCO_3_ (**c**) 0.2 mM displacing 1 mM KH_2_PO_4_ and (**d**) 1 mM displacing 0.2 mM KH_2_PO_4_. Convective effect is not included. The rate values are extracted at the interface between the two solutions.

The competitions between pH buffering and accumulation/depletion of H_3_O^+^ ions due to electromigative flux are shown in Fig. 6. When 0.2 mM displaces 1 mM NaHCO_3_, the accumulation rate of H_3_O^+^ ions is swiftly balanced by H_3_O^+^ depletion through the acid-base equilibrium reaction (see Equation S17 in Supplementary Note 2) to form HCO_3_^−^ ions (see Fig. 6a). Therefore, the net change of H_3_O^+^ is negligible (see Supplementary Fig. 2a) and the zeta potential is not altered significantly. Similarly (see Supplementary Fig. 2b), when the electric field is reversed, the depletion rate of H_3_O^+^ ions is being compensated by H_3_O^+^ production through the dissociation of HCO_3_^−^ ions to CO_3_^2−^ ions (see Fig. 6b).

For KH_2_PO_4_ solutions, the H_3_O^+^ ion depletion/accumulation rates due to electromigration and buffering reactions do not balance each other (see Fig. 6c,d). The net change of H_3_O^+^ ion concentration (see Supplementary Fig. 2c,d) causes a zeta potential difference between the two flow directions and generates noticeable EOF hysteresis. The simple buffer employed in this study is not optimized for pH buffering, but rather for demonstrating that the proper selection of buffer is crucial to suppress EOF hysteresis.

## Discussion

In this investigation, the origin of EOF hysteresis has been demonstrated in experiments and elucidated through numerical simulations. Although EOF is a surface phenomenon, rather counter-intuitively EOF hysteresis originates from the minority ionic behavior in the bulk of the fluid. The electric field gradient at the interface of two aqueous solutions generates electromigrative flux imbalance of minority pH-governing ions such as H_3_O^+^ ions and HCO_3_^−^ ions. These ions are thus accumulated or depleted at the interface. Subsequently, the ion depletion and accumulation zones widen and spread from the interface to the bulk of the solutions. The resultant pH change alters the zeta potential in the microchannel and the EOF flow rate, depending on the flow direction. When a high concentration/conductivity solution displaces a lower one, pH, zeta potential (magnitude) and flow rate are increased. On the contrary, when a lower concentration/conductivity solution displaces a higher one, pH, zeta potential (magnitude) and flow rate are decreased.

The EOF hysteresis can be visualized by plotting the average zeta potential (or flow rate) against the displacement of interface between the two solutions. The flow behaviors such as zeta potential, pH and flow rate can only be ascertained at a given interface position or time if the flow direction is known. EOF hysteresis can adversely affect the accuracy and consistency of flow control in microfluidic devices involving two or more fluids. Furthermore, the induced pH change can be detrimental to chemical and biological systems. It has been demonstrated that EOF hysteresis can be suppressed by careful selection of pH buffering solutions. The ion accumulation and depletion rate due to electromigration can be compensated by buffering acid-base reactions.

## Methods

### Preparation of solutions and microcapillaries

Three types of ionic solutions, namely KCl, NaHCO_3_ and KH_2_PO_4_ at concentration of 0.2 mM and 1 mM were prepared for the experiments. Stock solutions of 0.01 M were made by dissolving the salts (KCl from Fluka, NaHCO_3_ and KH_2_PO_4_ from Sigma-Aldrich) in deionized (DI) water. Thereafter, the stock solutions were diluted to concentrations of 0.2 mM and 1 mM. The solution properties, such as conductivity and pH, were measured with conductivity meter (IONCheck 65, Radiometer Analytical) and pH meter (FEP20, Mettler Toledo). The actual conductivity and pH measurements, as well as the numerical predictions, can be found in Supplementary Table 1.

Effect of Joule heating is insignificant because the conductivities of the solutions were low^25^. A conservative estimate of Joule heating can be calculated from the energy balance between the energy generation *E*_*g*_ and the energy storage ∆*E*_*st*_ in the liquid^26^. For the chosen experimental parameters, the worst case scenario has an estimated temperature rise of 0.11°C, which is negligible. Polyimide coated fused silica microcapillaries (Polymicro Technologies) with a nominal internal diameter of 100 μm and length of 8 cm were cut by Shortix Column Cutter (SGT Ltd). The microcapillaries were flushed with acetone followed by DI water, and lastly the experimental solutions before commencing the displacement flows.

The same microcapillary was used for the experiments of a particular solution pair in both flow directions. To ensure high accuracy and repeatability, the capillary was flushed with fresh solution through a syringe after each experimental run before it was used for the next run. This is a critical step to reset the pH and ionic condition in the capillary because the displacement flow process induces pH change. As a comparison, we have performed 4 additional runs with different pieces of capillary columns for the case of 1 mM KCl displacing 0.2 mM KCl. The average displacement time for these 4 runs is found to be 115.0 s with a standard deviation of 2.9 s. The average and standard deviation obtained from conducting experiments with different capillaries are not significantly different from the experimental run repeated with a single capillary (which is 118.1 s ± 3.9 s). The microcapillary was replaced with a new one for different solution pairs to avoid cross contamination.

At low Reynolds number (Re), the entrance/exit effect is negligible. For electro-osmotic flow, the dimensionless entrance length is approximately 0.11 Re^27^. For a microcapillary with 100 μm diameter (Re ~0.08), the entrance length is less than 1μm. The channel employed is 8 cm long. Therefore, the entrance/exit effect will not affect the experimental results. The thickness of EDL is given by the Debye length (for a symmetric electrolyte):


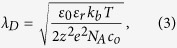


where *k*_*b*_ is the Boltzmann constant, *T* is the temperature, *z* is the absolute charge number of the main constituent ionic species, *e* is the electron charge, *N*_*A*_ is the Avogadro constant and *c*_*o*_ is the concentration of electrolyte solution. For our experiments, the Debye lengths are in the range of 10–30 nm. The diameter of microcapillary employed is 100 μm. Hence, there is no overlapping of EDL.

### Experimental setup and procedures

**Figure 7:**
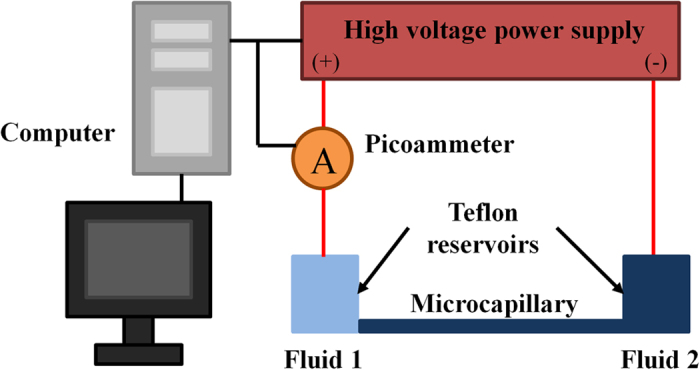
Current monitoring method. Schematic diagram of experiment setup for current monitoring method.

Current monitoring method is commonly employed to investigate the behavior of EOF^28^,^29^. Figure 7 shows the schematic diagram of the experimental setup employed for monitoring current change during two-fluid displacement flow. The electric field for inducing EOF was supplied by a high voltage power supply (CZE1000R, Spellman) through platinum electrodes. The current across the microchannel was monitored by connecting a picoammeter (Keithley 6458) in series to the microcapillary. A Labview program was written to control the two devices and to record the voltage and current readings through a data acquisition card (PCI-6052E, National Instrument). Two Teflon reservoirs with diameter and depth of 2 cm were fabricated. They were sufficiently large to ensure negligible liquid level changes during the experiment, thereby minimizes the back pressure generated due to the difference of liquid level in the reservoirs^30^. The application of large-volume reservoirs can also significantly dilute the concentrations of H^+^ and OH^−^ ions produced at the electrodes due to electrolysis^31^. Furthermore, the electrodes were positioned far from the inlet/outlet of the capillary to further prevent unwanted pH change in the microchannel due to electrolysis^32^.

The microcapillary and reservoir connecting to the cathode were filled with the solution to be displaced while reservoir connecting to the anode was filled with the displacing solution (Fig. 7). Electric potential of 1000 V was applied across the reservoirs to generate displacement flow of two opposing directions, i.e. 0.2 mM KCl displaces 1 mM KCl and 1 mM KCl displaces 0.2 mM KCl. These experiments were conducted for the three solution pairs. For every solution pair, displacement flows in two different directions were conducted five times each to ensure consistency and reliability of results. Zeta potentials of all the solutions employed were also measured via the current monitoring technique (see Supplementary Table 2 and Supplementary Note 1).

### Numerical model

**Figure 8:**
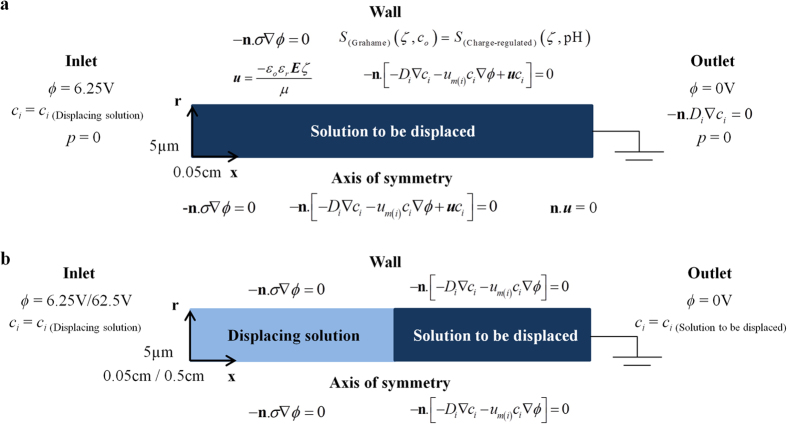
Numerical simulation domains, boundary and initial conditions. Numerical simulation domains, boundary and initial conditions for (**a**) full simulation (with convective effect) and (**b**) static simulation (without convective effect). **n** is the unit vector normal to the boundary.

Numerical studies were performed with finite element method (FEM) based on a modified slip-velocity model to elucidate the mechanism of EOF hysteresis. Slip-velocity model can be adopted because the microchannel diameter is much larger than the thickness of EDL (i.e. no overlapping EDL). Unlike the conventional slip-velocity model with constant zeta potential, the surface charge regulation model^33^ is incorporated to relate the zeta potential variation due to the deprotonation reaction of the silanol (SiOH) group on a microchannel surface. This wall boundary condition, together with Grahame equation (see Fig. 8a), can simulate the alteration of zeta potential due to the pH change induced by electromigrative flux imbalance of the minority pH-governing ions, as well as the concentration change of the main constituent ions (refer to charge-regulated Grahame equation in Supplementary Note 2). Comparison between the experimental and numerical zeta potentials validate the capability of our model in capturing the variation of zeta potential with solution properties (see Supplementary Table 2).

Reversible acid-base reactions are also included in our simulations (see Supplementary Note 2) because the concentrations of pH-governing ions are interdependent. The pH values in the numerical simulation are not prescribed *a priori* but obtained by solving the acid-base equilibria when the salt concentrations and equilibrium constants are known. The excellent agreement between the measured solution properties and numerical predictions validate the reliability and accuracy of our model in simulating the conductivity and pH of the solution (see Supplementary Table 1). The governing equations to formulate our newly-developed numerical model are:


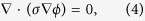



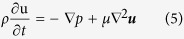



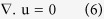



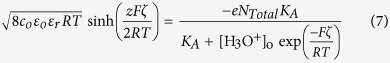



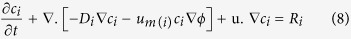


where *ϕ* is the applied electric potential, *ρ* is the fluid density, *p* is the pressure, ***u*** is the fluid velocity, *R* is the gas constant, *N*_*Total*_ is the total number of surface site density for SiOH, *K*_*A*_ is the equilibrium constant for deprononation of SiOH groups, [H_3_O^+^]_o_ is the bulk concentration of H_3_O^+^ ions, *D*_*i*_ is the diffusion coefficient and *R*_*i*_ is the overall reaction rate of each ionic species *i*. More details on the governing equations can be found in Supplementary Note 2.

The model assumes that the temperature of the solution is constant. Equilibrium constants, along with various solution properties such as viscosity and zeta potential are temperature dependant. To ensure negligible temperature rise, only low concentration solutions have been employed for our experiments. The temperature change due to Joule heating has been found to be negligible (see Experimental section).

### Simulation domains

The simulations were implemented on COMSOL Multiphysics software. The full model simulates the occurrence/suppression of EOF hysteresis to match the experimental observations (see Figs 1, 2a and Supplementary Fig. 1). Other simulations to demonstrate the mechanics of EOF hysteresis were performed without the convective term in Nernst-Planck equation, and convective-related governing equations (Navier-Stokes and continuity equations, see Supplementary Note 2).

The simulation domains are straight cylindrical channels with diameter of 10 μm and length of 0.05 cm or 0.5 cm (see Fig. 8). It is assumed that the center axis of the microchannel is axisymmetric. As the governing equations are strongly coupled (see Supplementary Note 2), high computational resources are required. Hence, the length of microchannel in the simulations was reduced as compared to the experiments. Since it had been shown that EOF hysteresis is independent of the voltage applied, length and diameter of the capillary^14^, scaled simulations provide good representations of the experimental flow behaviors.

### Boundary and initial conditions

The governing equations have to be solved simultaneously to obtain the time-dependent solution of two-fluid displacement flow. The initial conditions for the full simulation (with convective effect) were set to that of the solution to be displaced (see Fig. 8a). As for the simulation without flow, initial conditions of the left and right halves of the domain were set to the corresponding solution pair for the displacement flow (see Fig. 8b). The voltage at the inlet was set to establish an electric field of 125 V.cm^−1^ across the microchannel, which is similar to the experiments. The boundary conditions for the simulations with/without flow are as shown in Fig. 8. The symbols and values of parameters for the numerical simulations are given in Supplementary Table 3.

## Additional Information

**How to cite this article**: Lim, C. Y. *et al.* Ionic Origin of Electro-osmotic Flow Hysteresis. *Sci. Rep.* **6**, 22329; doi: 10.1038/srep22329 (2016).

## Supplementary Material

Supplementary Information
