# Supplementary material for: Ionic Origin of Electro-osmotic Flow Hysteresis
Source: Sci Rep. 2016 Feb 29;6:22329. doi: 10.1038/srep22329 (PMC4770316; doi:10.1038/srep22329)
Supplement: Supplementary Information [file srep22329-s1.doc]

**Supplementary Information**

**Ionic Origin of Electro-osmotic Flow Hysteresis**

Chun Yee Lim^1^, An Eng Lim^1^ & Yee Cheong Lam^1^

*^1^School of Mechanical and Aerospace Engineering, Nanyang Technological University, Nanyang Avenue 50, Singapore 639798*

**Supplementary Figures**

**
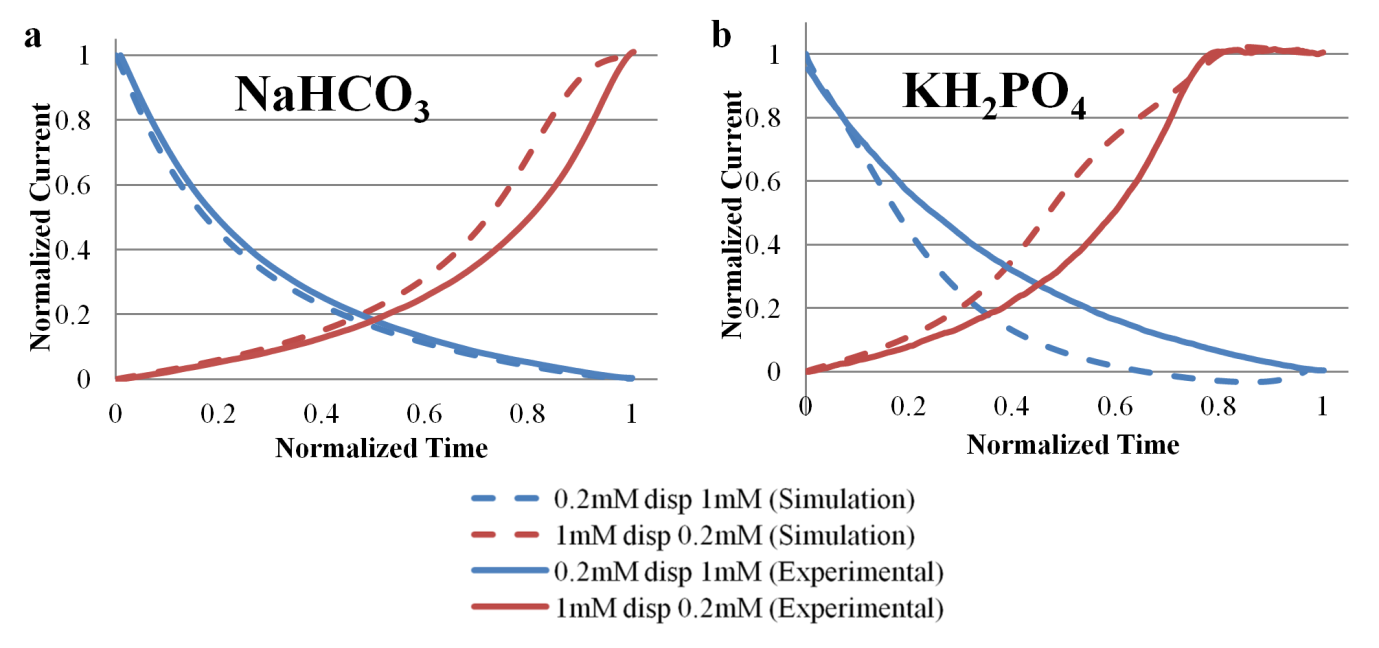
**

**Supplementary Figure 1 | Comparison between experimental and simulation results.** Experimental and simulated current-time curves during the displacement flow of 0.2mM and 1mM for (**a**) NaHCO_3_ (**b**) KH_2_PO_4_ solutions. Currents and displacement times are normalized with the two steady-state current values and time for 0.2mM to fully displace 1mM (*T_LH_*) respectively.


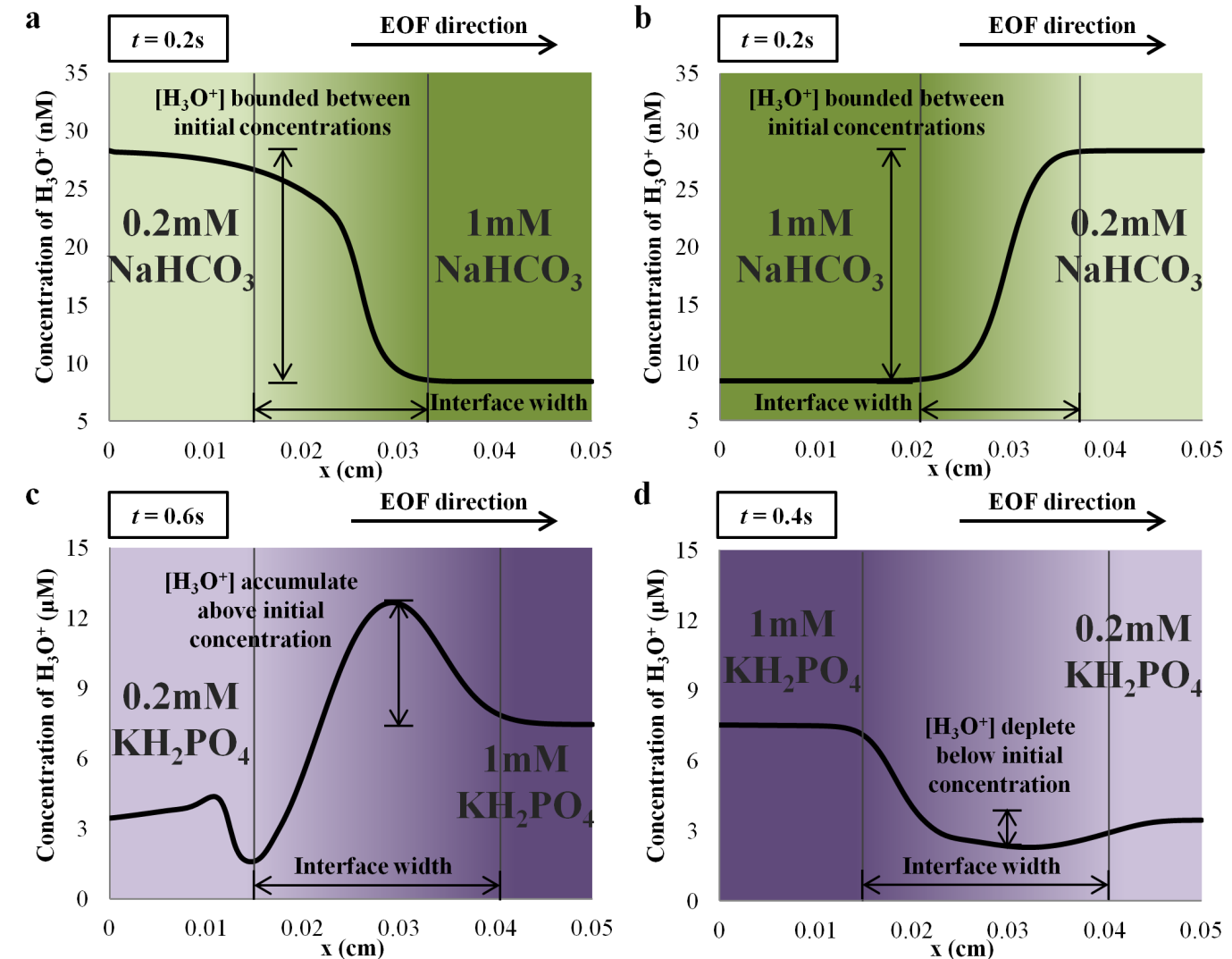


**Supplementary Figure 2 | Resultant H_3_O^+^ concentration from electromigration and buffering reactions during displacement flow.** Snapshots of simulated H_3_O^+^ concentration for NaHCO_3_ solution pair when (**a**) 0.2mM displaces 1mM (*t* = 0.2s) and (**b**) 1mM displaces 0.2mM (*t* = 0.2s), as well as for KH_2_PO_4_ solution pair when (**c**) 0.2mM displaces 1mM (*t* = 0.6s) and (**d**) 1mM displaces 0.2mM (*t* = 0.4s).

Supplementary Tables

| **Solution** | **Experimental** | | **Numerical** | |
| --- | --- | --- | --- | --- |
|  | **Conductivity (µS.cm^-1^)** | **pH** | **Conductivity (µS.cm^-1^)** | **pH** |
| 0.2mM KCl | 30.8 ± 0.1 | 5.62 ± 0.02 | 30.0 | 5.68 |
| 1mM KCl | 147.9 ± 0.1 | 5.73 ± 0.01 | 149.9 | 5.68 |
| 0.2mM NaHCO_3_ | 21.0 ± 0.1 | 7.49 ± 0.01 | 18.3 | 7.55 |
| 1mM NaHCO_3_ | 93.8 ± 0.3 | 7.90 ± 0.02 | 91.2 | 8.07 |
| 0.2mM KH_2_PO_4_ | 22.8 ± 0.2 | 5.53 ± 0.01 | 21.8 | 5.46 |
| 1mM KH_2_PO_4_ | 108.8 ± 0.1 | 5.19 ± 0.01 | 109.3 | 5.12 |

**Supplementary Table 1 | Conductivity and pH of solutions measured in experiments and employed in numerical predictions.**

| **Solution** | **Experimental**  **zeta potential (mV)** | **Numerical**  **zeta potential (mV)** |
| --- | --- | --- |
| 0.2mM KCl | -69.0 ± 1.7 | -67.0 |
| 1mM KCl | -51.6 ± 1.7 | -54.1 |
| 0.2mM NaHCO_3_ | -103.2 ± 3.3 | -139.1 |
| 1mM NaHCO_3_ | -117.2 ± 3.8 | -145.9 |
| 0.2mM KH_2_PO_4_ | -44.5 ± 2.9 | -58.8 |
| 1mM KH_2_PO_4_ | -33.5 ± 1.8 | -34.9 |

**Supplementary Table 2 | Experimental and numerical zeta potentials of solutions.**

| **Parameter** | **Symbol (Unit)** | **Value** |
| --- | --- | --- |
| Permittivity of free space | *ɛ_o_* (C.V^-1^.m^-1^) | 8.85 x 10^-12^ |
| Relative permittivity | *ɛ_r_* | 80 |
| Viscosity of water | *µ* (kg.m^-1^.s^-1^) | 8.90 x 10^-4^ |
| Density of water | *ρ* (kg.m^-3^) | 1000 |
| Faraday constant | *F* (C.mol^-1^) | 96485 |
| Gas constant | *R* (J.mol^-1^.K^-1^) | 8.314 |
| Temperature | *T* (K) | 298 |
| Electron charge | *e* (C) | 1.602 x 10^-19^ |
| Diffusion coefficient of K^+^ ion | *D_K_^+^* (m^2^.s^-1^) | 1.957 x 10^-9 (1)^ |
| Diffusion coefficient of Cl^-^ ion | *D_Cl_^-^* (m^2^.s^-1^) | 2.032 x 10^-9 (1)^ |
| Diffusion coefficient of Na^+^ ion | *D_Na_^+^* (m^2^.s^-1^) | 1.334 x 10^-9 (1)^ |
| Diffusion coefficient of H_3_O^+^ ion | *D_H3O_^+^* (m^2^.s^-1^) | 7 x 10^-10 (2)^ |
| Diffusion coefficient of OH^-^ ion | *D_OH_^-^* (m^2^.s^-1^) | 5.26 x 10^-9 (1)^ |
| Diffusion coefficient of H_2_CO_3_ | *D_H2CO3_* (m^2^.s^-1^) | 1.3 x 10^-9 (3)^ |
| Diffusion coefficient of HCO_3_^-^ ion | *D_HCO3_^-^* (m^2^.s^-1^) | 1.105 x 10^-9 (1)^ |
| Diffusion coefficient of CO_3_^2-^ ion | *D_CO3_^2-^* (m^2^.s^-1^) | 9.2 x 10^-10 (4)^ |
| Diffusion coefficient of H_3_PO_4_ | *D_H3PO4_* (m^2^.s^-1^) | 8.7 x 10^-10 (5)^ |
| Diffusion coefficient of H_2_PO_4_^-^ ion | *D_H2PO4_^-^* (m^2^.s^-1^) | 9.59 x 10^-10 (6)^ |
| Diffusion coefficient of HPO_4_^2-^ ion | *D_HPO4_^2-^* (m^2^.s^-1^) | 7.59 x 10^-10 (6)^ |
| Ionic mobility^a^ of K^+^ ion | *u_m(K_^+^)* (m^2^.V^-1^.s^-1^) | 7.621 x 10^-8^ |
| Ionic mobility of Cl^-^ ion | *u_m(Cl_^-^)* (m^2^.V^-1^.s^-1^) | -7.913 x 10^-8^ |
| Ionic mobility of Na^+^ ion | *u_m(Na_^+^)* (m^2^.V^-1^.s^-1^) | 5.195 x 10^-8^ |
| Ionic mobility of H_3_O^+^ ion | *u_m(H3O_^+^)* (m^2^.V^-1^.s^-1^) | 2.726 x 10^-8^ |
| Ionic mobility of OH^-^ ion | *u_m(OH_^-^)* (m^2^.V^-1^.s^-1^) | -2.048 x 10^-8^ |
| Ionic mobility of HCO_3_^-^ ion | *u_m(HCO3_^-^)* (m^2^.V^-1^.s^-1^) | -4.303 x 10^-8^ |
| Ionic mobility of CO_3_^2-^ ion | *u_m(CO3_^2-^)* (m^2^.V^-1^.s^-1^) | -7.166 x 10^-8^ |
| Ionic mobility of H_2_PO_4_^-^ ion | *u_m(H2PO4_^-^)* (m^2^.V^-1^.s^-1^) | -3.735 x 10^-8^ |
| Ionic mobility of HPO_4_^2-^ ion | *u_m(HPO4_^2-^)* (m^2^.V^-1^.s^-1^) | -5.912 x 10^-8^ |
| Ionic charge number of K^+^ ion | *z_K_^+^* | +1 |
| Ionic charge number of Cl^-^ ion | *z_Cl_^-^* | -1 |
| Ionic charge number of Na^+^ ion | *z_Na_^+^* | +1 |
| Ionic charge number of H_3_O^+^ ion | *z_H3O_^+^* | +1 |
| Ionic charge number of OH^-^ ion | *z_OH_^-^* | -1 |
| Ionic charge number of HCO_3_^-^ ion | *z_HCO3_^-^* | -1 |
| Ionic charge number of CO_3_^2-^ ion | *z_CO3_^2-^* | -2 |
| Ionic charge number of H_2_PO_4_^-^ ion | *z_H2PO4_^-^* | -1 |
| Ionic charge number of HPO_4_^2-^ ion | *z_HPO4_^2-^* | -2 |
| Equilibrium constant of SiOH deprotonation reaction | *K_A_* | 6.310 x 10^-8 (7,8)^ |
| Total number site density of SiOH | *N_Total_* (m^-2^) | 8 x 10^18 (8)^ |
| Apparent specific forward rate constant of water | *K_fw_* (mol.dm^-3^.s^-1^) | 1 x 10^-4 (9)^ |
| Water ionization constant | *K_w_* (mol^2^.dm^-6^) | 1 x 10^-14 (9)^ |
| Apparent specific forward rate constant of carbonate system 1 | *K_fC1_* (s^-1^) | 4.45 ^(9)^ |
| Equilibrium constant of  carbonate system 1 | *K_C1_* (mol.dm^-3^) | 4.45 x 10^-7 (9)^ |
| Apparent specific forward rate constant of carbonate system 2 | *K_fC2_* (s^-1^) | 0.469 ^(9)^ |
| Equilibrium constant of  carbonate system 2 | *K_C2_* (mol.dm^-3^) | 4.69 x 10^-11 (9)^ |
| Apparent specific forward rate constant of phosphate system 1 | *K_fP1_* (s^-1^) | 1 ^(b)^ |
| Equilibrium constant of  phosphate system 1 | *K_P1_* (mol.dm^-3^) | 7.5 x 10^-3 (9)^ |
| Apparent specific forward rate constant of phosphate system 2 | *K_fP2_* (s^-1^) | 1 x 10^-1 (b)^ |
| Equilibrium constant of  phosphate system 2 | *K_P2_* (mol.dm^-3^) | 6.2 x 10^-8 (9)^ |

^a^Ionic mobility of a specific ion species is calculated through the formula $\frac{z_{i}D_{i}F}{RT}$.

^b^Apparent specific forward rates for phosphate system are assumed to have the same order of magnitudes with carbonate system to reduce computational effort because the rates provided by Supplementary Reference 9 are too rapid. From a practical point of view, the assumption is reasonable as the exact values are of little importance. The reactions reach its equilibrium faster than other dynamic processes in the simulations^9^.

**Supplementary Table 3 | Symbols and values of parameters employed in numerical simulations.**

**Supplementary Note 1**

**Zeta potential measurement**

Zeta potential *ζ* of the experimental solutions were measured via the current monitoring technique (see Fig. 7 in main article). For the case of 1mM potassium chloride (KCl), the microcapillary and reservoir connecting to the cathode were filled with 1mM KCl while the reservoir connecting to the anode was filled with 0.95mM KCl (5% concentration difference). Electric potential of 1000V was applied across the two reservoirs to induce electro-osmotic flow (EOF). Experiments were conducted five times to ensure consistency and reliability of results. The time for the current to reach a steady value, i.e. the displacement time, was determined from the current-time curve (discussed in details later). The average electroosmotic velocity was then calculated by dividing the length of the channel with the displacement time. Thereafter, the zeta potential was obtained by substituting the average electroosmotic velocity into the Helmholtz-Smoluchowski slip velocity equation: *U* = *- ε_o_ε_r_Eζ* /*µ* , which can be expressed as

$\zeta=\frac{-\mu}{\varepsilon_{0}\varepsilon_{r}E}\cdot\frac{L}{T_{d}}$, (S1)

where *µ* is the viscosity of the liquid*, ε_o_* is the permittivity of free space, *ε_r_* is the relative permittivity of the liquid, *E* is the electric field, *L* is the length of the microchannel and *T_d_* is the displacement time.

**Determination of displacement time from current-time curve**

Electrical current in a cylindrical microcapillary is dependent on the applied field strength and conductivity of the solution. When a solution with a different conductivity (due to difference in ion concentration) flows into the capillary, the total resistance of the capillary is changed and this causes the current to change as well. The upstream and downstream fluids can be treated analogously as two variable resistors connected in series under a constant applied electric field with equivalent variable resistances written as

$\Re_{1}(t)=\frac{X(t)}{\sigma_{1}A}$, (S2)

$\Re_{2}(t)=\frac{L-X(t)}{\sigma_{2}A}$, (S3)

where $\Re_{i}(t)$ is the instantaneous resistance of fluid, *X*(*t*) is the interface displacement, *σ_i_* is the conductivity of fluid and *A* is the capillary cross-sectional area. With the assumption of a sharp interface between the two fluids, the time-dependent current^10^ according to Ohm’s law can be expressed as

$I(t)=\frac{V}{\Re_{T}(t)}=\frac{AV\sigma_{1}\sigma_{2}}{\left(\sigma_{2}-\sigma_{1}\right)X(t)+L\sigma_{1}}$, (S4)

where *V* is the applied electric potential and $\Re_{T}(t)$ is the total instantaneous resistance of the system.

To determine the displacement times from the experimental data, curve fitting of the current-time curves were performed through the regression analysis of SigmaPlot. Equation S4 can be re-written to facilitate curve fitting function in the following form:

$I^{-1}(t)=At^{n}+B$, (S5)

where *I^-1^(t)* is the inverse of experimental current, while *A*, *B* and *n* are the coefficients assigned for fitting. The R-squared values for the fittings were as high as 0.98, indicating the regression function is capable of capturing the experimental trend. Once *A*, *B* and *n* are determined, the displacement times were then calculated by evaluating *t* from Equation S5 when *I* is taken as the average current value upon completion of the displacement process.

**Supplementary Note 2**

**Laplace equation**

Application of an external electric field across the microchannel generates current. Assuming that there is no source or sink in the medium, charge conservation requires the divergence of current density ***J*** equals to zero,

$\nabla.\text{J}=0$. (S6)

Relationship between the current density ***J*** and transport of ions is given as

, (S7)

where the solution conductivity *σ* is defined as *F* ∑*z_i_u_m(i)_c_i_*, and *F* is the Faraday constant, *φ* is the applied electric potential, ***u*** is the fluid velocity, *u_m(i)_* is the ionic mobility, *z_i_* is the ion charge number, *D_i_* is the diffusion coefficient and *c_i_* is the concentration of each ionic species *i*. Equation S7 consists of the electromigrative, diffusive and convective currents whereby the first term is the major contributor; the other two terms are negligible due to their small magnitudes. Equation S7 is simplified to Laplace equation, which governs the applied electric potential *φ*,

. (S8)

**Navier-Stokes and continuity equations**

The fluid flow for an incompressible Newtonian fluid is governed by the Navier-Stokes and continuity equations in Equations S9 and S10,

, (S9)

$\nabla.\text{u}=0$, (S10)

where *ρ* is the fluid density, *p* is the pressure and *ρ_e_* is the net charge density. In microfluidics, the Reynolds number is typically less than 1, thus the inertial term (second term on left of Equation S9) is usually ignored and Stokes flow is assumed. Since the electrical double layer (EDL) thickness is small compared to the characteristic size of the experimental microchannel, flow inside the EDL is excluded in our numerical model. As such, we model only the flow of the bulk fluid (with zero net charge density) and the electric body force term (third term on right of Equation S9) is disregarded. To simulate EOF, electric field is obtained from the Laplace equation to specify the effective slip boundary condition (see Fig. 8a in main article) for solving the Navier-Stokes and continuity equations.

**Charge-regulated Grahame equation**

Zeta potential is an important parameter to characterize the direction and EOF velocity in a microchannel. During two-fluid displacement flow, the local zeta potential along the microchannel varies according to the local ion distributions. To capture this effect, a constant surface charge is prescribed on the channel wall, thus allowing zeta potential to be simulated based on the ion concentrations^11,12^. The surface charge density for symmetric electrolytes is determined by the Grahame equation

$S_{(\text{Grahame})}\left(\zeta,c_{o}\right)=\sqrt{8c_{o}\varepsilon_{o}\varepsilon_{r}RT}\text{ sinh}\left(\frac{zF\zeta}{2RT}\right)$, (S11)

where *c_o_* is the concentration of electrolyte solution, *R* is the gas constant, *T* is the temperature, *z* is the absolute charge number of main constituent ionic species and *e* is the electron charge.

EOF hysteresis originates from the accumulation/depletion of minority pH-governing ions such as hydronium ions (H_3_O^+^), as a result of electromigrative flux imbalance. Grahame equation does not reveal the changes of zeta potential in response to pH changes, which alters the EOF flow rate for different flow directions. To account for that, charge regulation^8^ due to deprotonation/protonation surface reactions occurring on the functional groups of the solid/liquid interface is incorporated to our numerical model. The deprotonation reaction of silanol (SiOH) groups; SiOH + H_2_O ⇌ SiO^-^ + H_3_O^+^, with equilibrium constant *K_A_* is assumed to occur when the glass/silica surface is placed in contact with the electrolyte solution. The protonation reaction of SiOH to produce SiOH_2_^+^ is expected only under extremely acidic conditions and will be disregarded^13^. The concentration of H_3_O^+^ ions at the solid/liquid interface follows the Boltzmann distribution. The relation between the surface charge density and charge-regulation is given as

$S_{(\text{Charge-regulated})}\left(\zeta,\text{pH}\right)=\frac{-eN_{Total}K_{A}}{K_{A}+[\text{H}_{3}\text{O}^{+}]_{\text{o}}\exp\left(\frac{-F\zeta}{RT}\right)}$, (S12)

where *N_Total_* is the total number of surface site density for SiOH and [H_3_O^+^]_o_ is the bulk concentration of H_3_O^+^ ions. Conjoining Equations S11 and S12 and setting it as the wall condition (see Fig. 8a in main article), gives zeta potential as functions of the solution concentration and pH.

**Reversible acid-base equilibria**

The minority pH-governing ions are dependent on one another via a set of reversible acid-base reactions. Our numerical model is formulated in terms of the kinetics of forward and reverse reactions for the dissociation of the weak acids/bases^9^. For example, consider the auto-ionization of water (H_2_O):

2H_2_O ⇌ OH^-^ + H_3_O^+^, (S13)

where OH^-^ represents the hydroxide ion. The rate of the forward reaction *r_f_* is

*r_f_* = *K_fw_* [H_2_O]^2^, (S14)

where *K_fw_* is the apparent specific rate constant for the forward reaction. The assumption that [H_2_O] does not change is made for this particular case. The rate of the reverse reaction *r_r_* is

*r_r_* = *K_rw_* [OH^-^] [H_3_O^+^] = *K_fw_ / K_w_* [OH^-^] [H_3_O^+^], (S15)

where *K_rw_* is the apparent specific rate constant for the reverse reaction, which can be derived by dividing *K_fw_* with the water ionization constant *K_w_*. With the forward and reverse reaction rates, the reaction rate *r* can then be computed and included into the Nernst-Planck equation to determine the concentration of a particular species. The apparent specific rate constants and equilibrium constants for the different weak acid-base chemical reactions are listed in Supplementary Table 3.

The existence of bicarbonate ions (HCO_3_^-^) in KCl solution is attributed to carbon dioxide (CO_2_) from the atmosphere dissolves in water. For this reason, water and carbonate systems are included for KCl solution, as well as sodium bicarbonate (NaHCO_3_) solution. The dissolved CO_2_ in equilibrium with the carbonic acid (H_2_CO_3_), represented by H_2_CO_3_*, under deprotonations through the acid-base equilibria:

H_2_CO_3_* + H_2_O ⇌ HCO_3_^-^ + H_3_O^+^, (S16)

HCO_3_^-^ + H_2_O ⇌ CO_3_^2-^ + H_3_O^+^. (S17)

For potassium dihydrogen phosphate (KH_2_PO_4_) solution, water and phosphate systems are considered. The triprotic phosphoric acid (H_3_PO_4_) can undergo deprotonation. To reduce the computational time, dissociation of hydrogen phosphate ion (HPO_4_^2-^) to phosphate (PO_4_^3-^) is removed as it is negligible within our pH range. The accuracy of the results is not compromised as shown by the excellent agreement between the experimental and numerical pH of KH_2_PO_4_ solutions (see Supplementary Table 1). The acid-base reactions employed are:

H_3_PO_4_ + H_2_O ⇌ H_2_PO_4_^-^ + H_3_O^+^, (S18)

H_2_PO_4_^-^ + H_2_O ⇌ HPO_4_^2-^ + H_3_O^+^, (S19)

where H_2_PO_4_^-^ represents the dihydrogen phosphate anion.

**Nernst-Planck equation**

The transports of all ionic species are simulated with the Nernst-Planck equation. The change of ion concentration with time is governed by the overall reaction rate and the gradients of three types of fluxes, namely diffusive, electromigrative and convective fluxes. Nernst-Planck equation can be written as

, (S20)

where *R_i_* is the overall reaction rate of each ionic species *i*. The overall reaction rate is defined as the summation of reaction rate *r* from the reversible acid-base chemical reactions.

**Supplementary References**

1. Newman, J. & Thomas-Alyea, K. E. *Electrochemical Systems*. (John Wiley & Sons, 2012).

2. Venkatnathan, A., Devanathan, R. & Dupuis, M. Atomistic simulations of hydrated nafion and temperature effects on hydronium ion mobility. *J. Phys. Chem. C* **111**, 7234-7244 (2007).

3. Nesic, S., Postlethwaite, J. & Olsen, S. An electrochemical model for prediction of corrosion of mild steel in aqueous carbon dioxide solutions. *Corros. Sci.* **52**, 280-294 (1996).

4. Zeebe, R. E. On the molecular diffusion coefficients of dissolved CO_2_, HCO_3_^-^, and CO_3_^2-^and their dependence on isotopic mass. *Geochim. Cosmochim. Acta* **75**, 2483-2498 (2011).

5. Leaist, D. G. Diffusion in dilute aqueous solutions of phosphoric acid. Verification of the limiting law for diffusion of weak electrolytes. *J. Chem. Soc., Faraday Trans. 1* **80**, 3041-3050 (1984).

6. Buffle, J., Zhang, Z. & Startchev, K. Metal flux and dynamic speciation at (bio) interfaces. Part I: Critical evaluation and compilation of physicochemical parameters for complexes with simple ligands and fulvic/humic substances. *Environ. Sci. Technol.* **41**, 7609-7620 (2007).

7. Leung, K., Nielsen, I. M. B. & Criscenti, L. J. Elucidating the bimodal acid-base behavior of the water-silica interface from first principles. *J. Amer. Chem. Soc.* **131**, 18358-18365 (2009).

8. Yeh, L. H., Xue, S., Joo, S. W., Qian, S. & Hsu, J. P. Field effect control of surface charge property and electroosmotic flow in nanofluidics. *J. Phys. Chem. C* **116**, 4209-4216 (2012).

9. Musvoto, E., Wentzel, M., Loewenthal, R. & Ekama, G. Integrated chemical–physical processes modelling—I. Development of a kinetic-based model for mixed weak acid/base systems. *Wat. Res.* **34**, 1857-1867 (2000).

10. Gan, H. Y., Yang, C., Wan, Y. M., Lim, G. C. & Lam, Y. C. Study of electroosmosis-driven two-liquid displacement flow in a microcapillary. *J. Phys. Conf. Ser.* **34**, 283-290 (2006).

11. Lim, C. Y. & Lam, Y. C. Direction dependence of displacement time for two-fluid electroosmotic flow. *Biomicrofluidics* **6**, 012816 (2012).

12. Lim, A. E., Lim, C. Y. & Lam, Y. C. Electroosmotic flow hysteresis for dissimilar ionic solutions. *Biomicrofluidics* **9**, 024113 (2015).

13. Behrens, S. H. & Grier, D. G. The charge of glass and silica surfaces. *J. Chem. Phys.* **115**, 6716-6721 (2001).
